# A comparison of sodium concentration measured in laboratory autoanalyser versus point-of-care blood gas machine: A retrospective, multicentre, analytical study in a large adult intensive care unit population

**DOI:** 10.1016/j.ccrj.2025.100149

**Published:** 2025-12-05

**Authors:** Keegan Hunter, Chris Anstey, Alexander Nesbitt, Karthik Venkatesh, Dinesh Parmar, Amanda Corley, Marissa Daniels, Jatinder Grewal, Kevin B. Laupland, Mahesh Ramanan, Alexis Tabah, James McCullough, Aashish Kumar, Antony G. Attokaran, Stephen Luke, Peter Garrett, Stephen Whebell, Sebastiaan Blank, Philippa McIlroy, Kyle C. White

**Affiliations:** a Intensive Care Unit, Princess Alexandra Hospital, Woolloongabba, Australia; b Professor, School of Medicine and Dentistry, Griffith University, Sunshine Coast Health Institute, Birtinya, Australia; c Mayne Academy of Critical Care, Faculty of Medicine, University of Queensland, St Lucia, Australia; d The George Institute for Global Health, Sydney, Australia; e Adult Intensive Care Services, The Prince Charles Hospital, Chermside, Australia; f Nursing and Midwifery Research Centre, Royal Brisbane and Women's Hospital, Herston, Australia; g School of Nursing and Midwifery, Griffith University, Australia; h The Prince Charles Hospital Northside Clinical Unit, Faculty of Medicine, The University of Queensland, Chermside, Australia; i Gatton Hospital, West Moreton Hospital and Health Service, Australia; j Caboolture Hospital, Caboolture, Australia; k Intensive Care Services, Royal Brisbane and Women's Hospital, Herston, Australia; l School of Clinical Sciences, Faculty of Health, Queensland University of Technology, Brisbane, Australia; m Critical Care Division, The George Institute for Global Health, University of New South Wales, Sydney, New South Wales, Australia; n Intensive Care Unit, Redcliffe Hospital, Redcliffe, Australia; o School of Medicine and Dentistry, Griffith University, Mount Gravatt, Australia; p Intensive Care Unit, Gold Coast University Hospital, Southport, Australia; q Intensive Care Unit, Logan Hospital, Logan, Australia; r Intensive Care Unit, Rockhampton Hospital, The Range, Australia; s Intensive Care Services, Mackay Base Hospital, Mackay, Australia; t College of Medicine and Dentistry, James Cook University, Townsville, Australia; u Intensive Care Unit, Sunshine Coast University Hospital, Birtinya, Australia; v Intensive Care Unit, Townsville University Hospital, Townsville, Australia; w Intensive Care Unit, Cairns Hospital, Cairns, Australia; x Intensive Care Unit, Queen Elizabeth II Jubilee Hospital, Coopers Plains, Australia

**Keywords:** Sodium concentration, ABG, Point-of-care, Laboratory, Autoanalyser, ICU, Critical care

## Abstract

**Objective:**

Discrepancies between laboratory sodium and point-of-care arterial blood gas sodium values may lead to delayed interpretation of, and intervention on, the results. We studied the mean difference between these two techniques and assessed the degree of agreement.

**Design:**

A multicentre, retrospective, observational study was conducted.

**Setting:**

Twelve intensive care units in Queensland, Australia, with tertiary-level hospitals accounting for 81% of admissions were included in the study.

**Participants:**

Adult patients with at least one paired laboratory sodium and arterial blood gas measurement during their intensive care unit admission were a part of this study.

**Main outcome measures:**

Main outcome measures included mean difference between laboratory sodium and point-of-care sodium measurement, with a positive difference demonstrating laboratory sodium values higher than arterial blood gas sodium values.

**Results:**

A total of 65,042 patients with 224,383 paired samples were included in the analysis. The Bland–Altman mean difference of laboratory sodium and arterial blood gas sodium was 0.72 mmol/L (95% limit of agreement [LoA]: 4.35) with a Deming regression slope of 0.93 (95% confidence interval: 0.92, 0.94) and intercept +10.07 (p < 0.001). On subgroup analysis of hyponatraemia, eunatraemia and hypernatraemia a mean difference (95% LoA) of 1.53 mmol/L (4.21), 0.15 mmol/L (4.39), and −1.02 mmol/L (5.37), was calculated, respectively. Patients with severe hyperglycaemia and normal albumin had a mean difference (95% LoA) of −1.85 mmol/L (4.78). Analysis of mild, moderate, and severe subgroups within both hyponatraemic and hypernatraemic samples showed increasing mean differences, with severe hyponatraemia showing a mean difference of 2.01 mmol/L (95% LoA: 8.08) and severe hypernatraemia showing a mean difference of −4.7 mmol/L (95% LoA: 15.46).

**Conclusions:**

Point-of-care arterial blood gas sodium measurements show small mean differences in eunatraemia and good agreement with paired laboratory samples in adult intensive care unit patients. Caution should be applied when interchanging results between laboratory and point-of-care sodium values in patients with moderate to severe dysnatraemia, as serial measurements using different methods during treatment are unlikely to be within a clinically acceptable range. This is important when caring for patient groups with severe hyponatraemia and induced hypernatraemia, and serial measurement may be better achieved with point-of-care testing due to a combination of ease of access, repeatability, and lower cost.

**Key points**

i) Laboratory sodium measurement and point-of-care machines show good agreement and small mean differences in eunatraemia but larger differences that are likely to have clinically meaningful differences in severe dysnatraemia.
ii) Laboratory sodium measurements tend to be higher than point-of-care results when hyponatraemia is present.
iii) Laboratory sodium measurements tend to be lower than point-of-care results when hypernatraemia is present.
iv) In the clinical setting of repeated testing with a single modality, the benefits of point-of-care testing to guide sodium correction are likely the superior choice for clinicians.

## 1 Introduction

Sodium (Na) is the primary extracellular cation and the major contributor to extracellular space tonicity, with a normal concentration (eunatraemia) of 135–145 mmol/L.^1^ Dysnatraemia has numerous causes and may be classified by the degree of change, the plasma tonicity, and patient volume status.^2^^,^^3^ Hyponatraemia (Na <135) and hypernatraemia (Na >145) are common issues in intensive care unit (ICU) patients, and each is associated with increased mortality.^2^^,^^4^^,^^5^ Therefore, maintenance and management of plasma sodium levels is a core part of intensive care practice, particularly in patients with traumatic brain injury (TBI) or in patients with severe dysnatraemia as their primary presentation.^6^^,^^7^ Sodium concentration is routinely measured in clinical practice, with measurements via point-of-care (POC) arterial blood gas (ABG) machines or central laboratory autoanalysers.

Blood gas analysis is an accurate, rapid, and cost-effective method for assessing electrolytes, partial pressures of oxygen and carbon dioxide, and important metabolites in the ICU.^8^^,^^9^ Arterial catheters are routinely inserted in patients admitted to Australian ICUs, allowing repeated blood sampling for ABG analysis.^10^ Measurement of sodium concentration in a blood gas analyser uses a direct ion-selective electrode (ISE) method, measuring sodium activity in the whole blood sample across the electrochemical sensor.

Laboratory autoanalysers are designed for high-throughput assays, using indirect ISEs, and can run multiple tests on a single serum sample. Serum samples require an approximate 30-min standing time, which introduces a significant delay in processing and analysis. Lithium heparin tubes allow faster analysis of centrifuged plasma; however, they are still subject to delays in blood-sample transport, laboratory reception and sorting, and preprocessing requirements.^11^ Indirect ISE methods rely on premeasurement dilution of centrifuged whole blood, using standardised values for the nonaqueous phase of blood. They are susceptible to error when the lipid or protein content of the sample varies.^12^

Previous studies have shown discordance in values of agreement between laboratory and POC sodium measurements, although they have been small, with inconsistent methods and varied patient populations.[13], [14], [15], [16], [17], [18] The increased risk of adverse outcomes related to dysnatraemia in the ICU, prevention of secondary brain injury in specific cohorts, and the high frequency of sodium measurements performed on both POC and laboratory samples requires consistent measurements of serum sodium. The benefits of each method inform use in different scenarios; however, there is equipoise in whether the results of each method are routinely interchangeable. Accordingly, we investigated whether POC ABG–derived sodium measurements were comparable to laboratory measurements and suitable to inform decision-making in clinical practice, determined in a diverse cohort of critically unwell adults admitted to ICUs in Australia.

## 2 Methods

### 2.1 Study design and setting

This retrospective analytical study utilised data from adult (≥18 years old) patients admitted to 12 participating ICUs in Queensland, Australia, from 2015 to 2021. These ICUs included most of the State’s tertiary cardiothoracic, neurosurgical, and trauma ICUs, along with other mixed medical and surgical ICUs.

### 2.2 Data collection

Data were sourced from a database linking Queensland Pathology to MetaVision (iMDsoft) ICU admissions and the Australian and New Zealand Intensive Care Society Adult Patient Database for diagnostic codes and diagnostic groups. All ABG samples were completed on Radiometer ABL (Denmark) machines, and the laboratory sample was processed on Beckman Coulter (California) general chemistry analysers with either the DxC800 or DxC600 model machines in each hospital. All ABG and laboratory autoanalyser machines were internally and remotely calibrated according to Queensland pathology guidelines.

### 2.3 Paired samples

All sodium measurements were assessed for pairing, and the first paired sample of the day was included from every patient over the entire study period. Measurements were considered paired when the POC and laboratory samples were collected in the same hour. Sodium values <100 mmol/L or >180 mmol/L were excluded from the analysis due to the small sample number. There were no clinical exclusion criteria.

Hyponatraemia was classified into mild (130–134 mmol/L), moderate (120–129 mmol/L), or severe (Na <120 mmol/L) (8, 9, 12). Hypernatraemia was classified into mild (146–150 mmol/L), moderate (151–160 mmol/L), and severe (Na >160 mmol/L). TBI was identified using the diagnostic group within the Australian and New Zealand Intensive Care Society Adult Patient Database Data Dictionary.

### 2.4 Outcomes

The primary outcome was the mean difference between laboratory sodium (NaL) and POC sodium (NaP) concentrations. The difference was calculated using the formula laboratory sodium minus POC sodium value. The secondary outcome was the calculated mean difference in different severity subgroups of hyponatraemia and hypernatraemia, and assessment of biochemical factors that were independently associated with the difference between sodium values according to the two methods. These included albumin level, glucose level, lactate, and pH. Albumin level was classified as normal (>40–60 g/L), low (20–40 g/L), and severely low (<20 g/L). Glucose level was dichotomised to severely elevated (>20 mmol/L) and nonsevere levels (<20 mmol/L). The exploratory outcome was the incidence of a difference in dysnatraemia classification between paired samples using either laboratory or POC measurement.

### 2.5 Statistical methods

A Bland–Altman plot was constructed to compare the mean difference of each paired sample between the two sodium measurement methods. The agreement between sodium measurements was assessed with Deming regression analysis as both measurement methods were assumed to have inherent measurement error. A post hoc regression analysis of the difference to the mean was assessed in the whole group and subgroups of hyponatraemia, eunatraemia, and hypernatraemia to assess for proportional bias. Intraclass correlation coefficients (ICCs) (absolute agreement) using a two-way mixed-effect model were calculated. Descriptive statistics were expressed as means with standard deviations (SDs), medians with interquartile ranges, and frequencies and proportions for categorical variables. A mixed-effect linear regression model, including the individual patient as a random effect, was developed to examine which biochemical variables were independently associated with the difference between POC and laboratory methods. Variables in the analysis were decided a priori and included those often severely deranged in critically ill patients: glucose, albumin, lactate, urea, chloride, and pH. The results of the multivariable analysis were reported as coefficients with 95% confidence intervals (95% CIs). Statistical analyses were performed using STATA™ (StataCorp LLC) (version 17.0). P values <0.01 were considered significant. A sensitivity analysis was performed using a random sample for each patient to assess whether multiple measurements from patients would meaningfully affect the assumption of independence in the Bland–Altman analysis.

### 2.6 Ethical consideration

Data collection and use in this study were approved by the Metro South Hospital and Health Service Human Research Ethics Committee (HREC/2022/QMS/82024), and an individual waiver of consent was granted.

## 3 Results

### 3.1 Patient characteristics

The database yielded 65,042 unique patients (mean age: 61 years, female sex: 38%) and 224,383 paired samples with a mean of 3.45 samples/patient. The most common Acute Physiology and Chronic Health Evaluation IIIJ diagnosis groups were cardiovascular (34%), gastrointestinal (13%), and neurological (13%) (Table 1). Admission source was most commonly from operating theatres (57%) and emergency departments (EDs) (26%). The study population represented all Acute Physiology and Chronic Health Evaluation IIIJ diagnostic groups.

**Table 1:** Population characteristics.

Characteristic (N = 65,042)	
Age (years)	61.0 (48.0, 71.0)
Female	24,576 (38%)
APACHE^a^ diagnosis group (top 6)	
Cardiovascular	22,251 (34%)
Gastrointestinal	8499 (13%)
Neurological	8284 (13%)
Respiratory	7191 (11%)
Sepsis	5480 (8.4%)
Trauma	4894 (7.5%)
Admission circumstances	
Post-elective surgery	26,425 (41%)
Length of stay in hospital before ICU (hours)	12.42 (4.50, 47.23)
ICU level	
Tertiary	52,866 (81%)
Source of ICU admission	
Emergency department	16,242 (26%)
Operating theatre	36,313 (57%)
Ward	8060 (13%)
Other hospital	3008 (4.7%)

a APACHE: Acute Physiology and Chronic Health Evaluation; ICU: intensive care unit.

### 3.2 Primary outcome

**Fig. 1:**
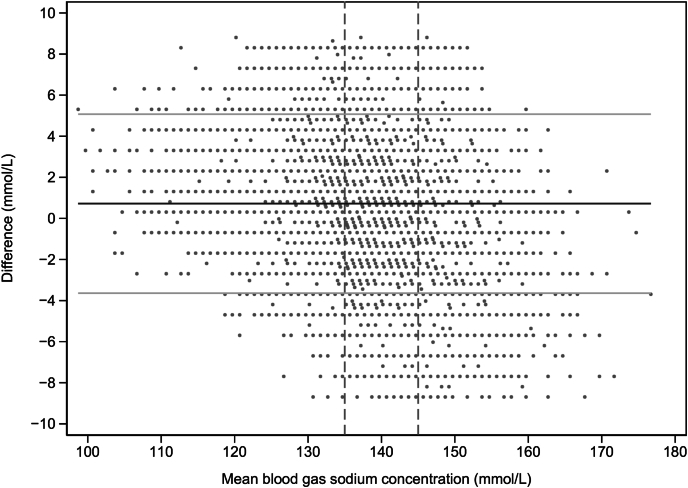
Bland–Altman plot. The horizontal solid black line indicates the mean difference (95% CI) 0.72 mmol/L (−3.63 and 5.07 mmol/L). The normal sodium range is marked by the vertical dashed lines. For clarity, differences greater than ±4 SD are not shown. CI: confidence interval.

**Fig. 2:**
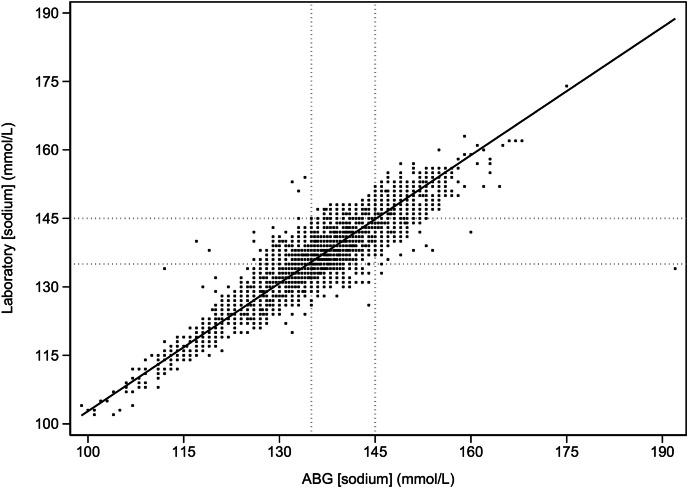
Deming regression model for all data, where slope (b) = 0.93 (95% CI: 0.92, 0.94), intercept (c) +10.07 (p < 0.001), where NaL = NaP∗b + c. ABG: arterial blood gas; CI: confidence interval.

The mean difference (95% limit of agreement [LoA]) of sodium concentration in the paired samples was 0.72 mmol/L (−3.36 to +5.07 mmol/L) (Fig. 1). The mean sodium concentration (SD) in laboratory samples was 138.14 mmol/L (5.01), and the mean sodium concentration (SD) in POC ABG samples was 137.42 (5.31). That is, laboratory sodium values averaged 0.72 mmol/L above ABG measurements, with a median (interquartile range) of 0 mmol/L (−1.0, 2.0) and a mode of 0 mmol/L (electronic supplementary material [ESM] Fig. 1). An absolute difference ≤4 mmol/L was seen in 94.2% of samples. The Deming regression analysis showed a slope of 0.93 (0.92, 0.94) and an intercept of +10.07 (p < 0.001) (Fig. 2). There was significant proportional bias, with a slope of −0.23 (−0.24, −0.21) and an intercept of 136.4, p < 0.001. Sample predictions across all subgroups are provided in the ESM (ESM table 1). The ICC for individual results across all data was 0.83 (0.82, 0.85) p < 0.001 (ESM table 2).

### 3.3 Sodium subgroups

There were 56,931 samples within the hyponatraemia group, and the mean difference (95% LoA) was 1.53 mmol/L (4.21). There were 149,469 samples in the eunatraemia group and a mean difference (95% LoA) of 0.15 mmol/L (4.39). There were 17,983 samples in the hypernatraemia group and a mean difference (95% LoA) of −1.02 mmol/L (5.37) (Table 2). Significant proportional bias was present in each of these subgroups with slope and intercept values of +0.33 (+0.31, +0.35) and 132.1, +0.18 (+0.17, +0.19) and 138.0, and +0.17 (+0.12, +0.23) and 148.0 in hyponatraemia, eunatraemia, and hypernatraemia groups, respectively.

**Table 2:** Subgroup analysis of mean difference of laboratory sodium and ABG sodium.

Group	N	Mean difference mmol/L (95% LoA^a^)
Hyponatraemia	56,931	+1.53 (4.21)
Eunatraemia	149,469	+0.15 (4.39)
Hypernatraemia	17,983	−1.02 (5.37)

Mean difference is equal to laboratory sodium minus POC sodium value.

a ABG: arterial blood gas; LoA: limit of agreement; POC: point of care.

Two clinical groups, hyponatraemia and hypernatraemia, were each further analysed in mild, moderate, and severe subgroups (section 2.3). Samples with mild, moderate, and severe hyponatraemia had calculated mean differences (95% LoA) of 1.45 mmol/L (4.08), 1.99 mmol/L (4.51), and 2.01 mmol/L (8.08), respectively. Likewise, mild, moderate, and severe hypernatraemia samples had calculated mean differences (95% LoA) of −0.77 mmol/L (4.88), −1.42 mmol/L (5.57), and −4.7 mmol/L (15.46), respectively (ESM table 3).

A reanalysis of all POC sodium values compared to laboratory sodium value resulted in less than 0.05% (n = 7) of samples classed as hypernatraemia being reclassified into the eunatraemia group. POC measurement led to reclassification of 6.43% (n = 10,271) laboratory hyponatraemia samples to eunatraemia (Table 3). The McNemar–Bowker and Stuart–Maxwell tests for symmetry and marginal homogeneity, respectively, were significant across all subgroups (p < 0.001) (ESM table 5).

**Table 3:** Classification of sodium value as hypernatraemia, eunatraemia, and hyponatraemia based on laboratory or POC value.

Characteristic	Laboratory sodium N = 224,383 (%)	POC sodium N = 224,383 (%)	p value
Subgroup			<0.001
Hyponatraemia	56931 (25.4%)	46660 (20.8%)	
Eunatraemia	149469 (66.6%)	159733 (71.2%)	
Hypernatraemia	17,983 (8.0%)	17,990 (8.0%)	

POC: point of care.

### 3.4 Clinical subgroups

A subgroup analysis in all patients with a coded diagnosis of TBI showed that patients with TBI had a mean difference (95% LoA) of 0.62 mmol/L (4.31) between NaL and NaP and higher mean values than non-TBI patients in both NaL (140.2 vs 137.9 mmol/L, p < 0.001) and NaP (139.6 vs 137.2 mmol/L, p < 0.001) values (Table 4).

**Table 4:** Mean sodium value with stratification by TBI.

Subgroup	Laboratory sodium (SD^a^) (mmol/L)	POC sodium (SD) [mmol/L]	Mean difference (95% LoA) [mmol/L]
TBI	140.2 (5.4)	139.6 (5.5)	+0.62 (4.31)
Non-TBI	137.9 (4.9)	137.2 (5.1)	+0.72 (4.47)
p-values	<0.001	<0.001	<0.001

LoA: limit of agreement; POC: point of care; TBI: traumatic brain injury.

a – standard deviation.

An analysis in patients with a blood glucose level (BGL) > 20 mmol/L showed a mean difference (95% LoA) of −0.12 (6.02), compared to +0.73 (4.43) in patients with a BGL <20 mmol/L (p < 0.001). Stratification by BGL result and albumin level showed a maximal mean difference −1.85 (p < 0.001) in patients with a BGL >20 mmol/L and albumin >40 g/L. Exclusion of all results with a BGL >20 or albumin <20 g/L showed a mean difference (95% LoA) 0.66 mmol/L (4.43) (Table 5).

**Table 5:** Stratification by albumin level and blood glucose level.

Mean difference (mmol/L) (95% limit of agreement)					
Subgroup	All albumin levels	Albumin >20 g/L	Albumin <20 g/L	20 g/L ≤ albumin ≤40 g/L	Albumin >40–60 g/L
Blood glucose level (BGL) <20 mmol/L (n = 169,908)		+0.66 (4.43)			
BGL ≤20 mmol/L (n = 197,194)	+0.73 (4.43)		+1.27 (4.31)	+0.66 (4.43)	−0.41 (4.70)
BGL >20 mmol/L (n = 1918)	−0.12 (6.02)		+1.03 (5.61)	−0.26 (6.02)	−1.85 (4.78)

Values are mean difference in measured sodium value (SD). 20 mmol/L = 360 mg/dL.

### 3.5 Multivariable regression

Multivariable regression for clinically significant factors on the routine ABG measurement was assessed. The effect of pH in 0.10 increments (0.133 [95% CI: 0.120, 0.145]; p < 0.001), chloride (−0.027 [95% CI: -0.028, −0.025]; p < 0.001), and albumin (−0.048 [95% CI: -0.049, −0.046]; p < 0.001) was independently associated with the difference between the two measurements (ESM table 6). Urea and lactate did not show a significant association (p > 0.01).

### 3.6 Sensitivity analysis

A sensitivity analysis with a random sample for each patient showed that both datasets were normally distributed and that the mean difference (95% LoA) for a random sample was +0.17 (4.70), compared to all data +0.72 (4.35) (p < 0.001) (ESM table 7).

## 4 Discussion

### 4.1 Key findings

In this multicentre study of more than 65,000 critically ill patients and over 224,000 paired sodium measurements, we showed that laboratory and POC ABG results had small mean differences and a high level of agreement in a mixed ICU population. Eunatraemic paired samples had values close to parity, and most paired samples had a difference of zero. Hyponatraemic samples averaged greater values when measured by laboratory methods, and hypernatraemic samples averaged greater values when measured by POC ABG methods. An increasing mean difference with increasing severity of both hyponatraemia and hypernatraemia suggests that serial measurements in severe dysnatraemia, using different methods during treatment, are unlikely to be clinically comparable. This is supported by evidence of proportional bias in each subgroup, as well as lower ICC values in the hyponatraemia and hypernatraemia subgroups. The higher ICC for all data may be due to a disproportionately greater number of eunatraemic samples with a narrower CI. POC testing diagnosed less hyponatraemia, though an absolute difference of less than 4 mmol/L was observed in 94.2% of all samples, indicating that most reclassification was mild, and while certain complications (altered cognition, seizure, and coma) are related to the severity of dysnatraemia, this reclassification bias is less clinically relevant for clinical care as guidelines on treatment are primarily focused on the absolute value, and absolute change, rather than the severity per se . Reassuringly, other common biochemical factors including pH, lactate, and urea did not have a clinically important effect on the variability between the sodium measurements.

In patients with TBI, an important clinical subgroup that often receives exogenous therapeutic sodium, which is typically checked repeatedly after intervention, the mean difference remained clinically comparable. The average sodium value was higher in both NaL and NaP groups, likely indicating therapeutic intent and adherence to international guidelines.^6^ Albumin and severe hyperglycaemia showed an important effect, with the largest difference occurring in patients with severe hyperglycaemia and normal albumin levels. While the overall mean difference in the severely hyperglycaemic group was small, there was a clear effect when stratifying by albumin level. This glucose effect has been observed before^19^ and may represent method-dependent variation beyond water movement and the physiological effects of hyperglycaemia.^20^ When albumin was low, in both nonsevere and severe hyperglycaemic samples, laboratory sodium measured higher than POC sodium, consistent with a mild pseudohypernatraemia effect.

### 4.2 Relation to literature

The agreement of NaL to NaP has been investigated before, and the outcome has differed based on sample size, sampling type, and hospital patient group. An Australian ICU sample reported a mean sodium difference of 1.49 mmol/L in a similar population of adult ICU patients.^9^ A UK study in a mixed adult and paediatric ICU cohort reported a mean absolute difference of 0.57 mmol/L and a Pearson correlation coefficient of 0.94.^15^ While these were well-designed studies in ICU patients, both were smaller, n = 219 and n = 9068, while the UK group allowed either arterial or venous samples collected in a mixed paediatric and adult population. Neither the interaction in subgroups nor the effect of metabolites was assessed. Our findings showed a mean difference comparable to these two studies; however, our large sample size provides additional data in dysnatraemia, and this challenges conclusions from these two studies that NaP is clinically comparable to NaL, outside the normal range.

Other investigators have concluded that regardless of the measured sodium level, laboratory results and blood gas machine results cannot be interpreted as clinically interchangeable. Significant methodological differences include much smaller sample sizes in all identified studies[13], [14], [15], [16], [17], [18]^,^^21^ and including large numbers of patients presenting to the ED. Solak^14^ compared NaL and NaP in three similar subgroups in 2557 paired samples in an ED population. They found absolute mean differences (SD) of 4.4 (4.7), 4.5 (3.7), and 5.9 (4.2) mmol/L. Despite similar analysers and POC equipment, these values significantly exceed our own. Delays in the collection, processing, and varying collection methods, such as venepuncture, may explain these differences. Mirzazadeh et al. previously reported smaller mean differences in an ICU versus ED population.^15^

Hospital samples (including ICU) measured by laboratory analysers had previously shown to overestimate sodium concentration when total protein (TP) was low and underestimate sodium concentration when TP was high.^22^ Although our results used albumin level as a surrogate for TP, as TP was not available in our study, the association was similar.

## 5 Implications of findings

Our findings show that across a normal sodium range, the mean difference between NaL and NaP is small; however, limits of agreements are wide and likely to involve a clinically meaningful range. This effect was further sustained in the subgroup analysis, and the more severe the dysnatraemia, the less comparable the results. This may challenge the belief that a “once-daily” laboratory check of sodium to ensure ABG “accuracy” is necessary. The effect of glucose and albumin levels was inconsistent, and this is likely due to the surrogate nature of albumin for TP, and BGL in the analysis as a dichotomous variable. Nonetheless, the absolute mean difference was less than 2 mmol/L and in the absence of severe dysnatraemia, may not represent a clinically significant variation.

Guidelines currently suggest sodium correction in high-risk patients with moderate to severe hyponatraemia should be limited to 6–8 mmol/L/day,^2^^,^^23^ though recent evidence challenges previous approaches to slow sodium correction in severe hyponatraemia^24^ and hypernatraemia.^25^ Even so, in severe hyponatraemia, our calculated mean difference (95% LoA) of 2.01 mmol/L (8.08) may prevent interchanging results between sampling methods in this specific population as it may lead to rapid overcorrection. Patients with TBI may have sodium targets 145–155 mmol/L, and in this range, the mean difference was less than 2 mmol/L, although with 95% LoA up to 5.57 mmol/L, interchanging the results may not be acceptable. In severe hypernatraemia, a calculated mean difference of −4.70 mmol/L is clinically significant, though this was limited by the relatively small sample number and its significance reduced by the less-established harms of overcorrection in adults. This study did not assess the accuracy or repeatability of each method, and thus it is not possible to draw conclusions on which is more accurate for showing a trend over time. In ICU patients requiring frequent sodium measurement or correction, POC machines provide the benefits of rapid and repeatable testing while also remaining cost-effective.^9^

## 6 Strengths and limitations

The limitations of this study relate to its retrospective nature and inability to match samples to less than 1-h separation. This was addressed by excluding paired samples with more than two POC or two laboratory samples within the hour. However, it is possible that therapeutic interventions such as an urgent, exogenous sodium load or utilisation of medications to modify aquaresis and natriuresis were undertaken after one sample was taken and before the second (paired) sample was taken to assess the response, particularly in the TBI cohort. Reassuringly, the mean difference and standard deviation were comparable between the TBI and non-TBI cohorts. Determination of Bland–Altman mean difference relies on the assumption of independent measurements, and it is possible that the daily measurements from each patient had unidentified variables that violate this assumption. However, our sensitivity analysis showed a 95% LoA of ±4.70 mmol/L, which is comparable to that of the whole dataset, 4.35 mmol/L. Dry heparinised electrolyte–balanced blood gas syringes can lead to errors in sodium concentration, especially if small volumes of blood are sampled.^26^ This source of error was expected to be mitigated with standardised nursing training and the collection of sufficient volume from arterial catheter sampling. Our data were collected from ICUs encompassing both smaller, non–tertiary level centres and those managing cardiothoracic, neurosurgical, and trauma patients. This may limit the generalisability to these specific cohorts; however, it reflects routine clinical practice with mixed patient cohorts and improves external validity. This study did not try to compare the accuracy of these different techniques of measuring sodium concentration because neither was considered a gold standard reference. Thus, despite being reported previously,^14^ reference was not made to United States Clinical Laboratory Improvement Amendments (USCLIA) standards or the anticipated accuracy of each methodology.^9^ As collection technique and laboratory processing were conducted per local practice, further investigation would benefit from prospectively time-matched samples and a protocolised collection and processing technique to reduce errors associated with delays to measurement.

The study’s significant strengths include its large sample size, longitudinal data collection throughout each patient’s ICU admission, and subgroup analysis of clinically relevant patient subgroups. The highly granular data and explanatory analysis on the lack of clinically significant effect from commonly deranged biochemical markers should increase a clinician’s confidence in being informed and acting on early POC results.

## 7 Conclusions

This observational study shows that for critically ill patients managed in the ICU, the level of agreement between laboratory autoanalyser and POC ABG sodium measurement varies and appears to be dependent on the presence and severity of dysnatraemia. Eunatraemic samples are sufficiently similar and allow immediate interpretation and intervention. Due to the apparent divergence of NaL and NaP with increasing severity of dysnatraemia, the benefits of POC testing suggest it remains the method of choice when treating or monitoring moderate–severe dysnatraemia using serial sampling.

## Statement of ethics

The Metro South Hospital and Health Service Human Research Ethics Committee (HREC/2022/QMS/82024) approved this study, and an individual waiver of consent was granted.

## CRediT authorship contribution statement

The study conception and design (all authors); data acquisition (KW); analysis (CA, KH, and KW); interpretation of data (all authors); article draughting (CA, KH, MR, and KW); article revision for important intellectual content (all authors); final approval of the version submitted for publication (all authors); agreement to be accountable for all aspects of the work in ensuring that questions related to the accuracy or integrity of any part of the work are appropriately investigated and resolved (all authors).

## Data availability statement

Data cannot be shared publicly due to institutional ethics, privacy, and confidentiality regulations. Data released for research under Sect. 280 of the Public Health Act 2005 require an application to the Director-General of Queensland Health (PHA@health.qld.gov.au).

## Funding

MR acknowledges support from the Metro North Hospital and Health Services Clinician-Research Fellowship.

## Conflict of interest

The authors have no conflicts of interest to declare.
