# Supplementary material for: A comparison of sodium concentration measured in laboratory autoanalyser versus point-of-care blood gas machine: A retrospective, multicentre, analytical study in a large adult intensive care unit population
Source: Crit Care Resusc. 2025 Dec 5;27(4):100149. doi: 10.1016/j.ccrj.2025.100149 (PMC12723123; doi:10.1016/j.ccrj.2025.100149)
Supplement: Multimedia component 1 [file mmc1.docx]

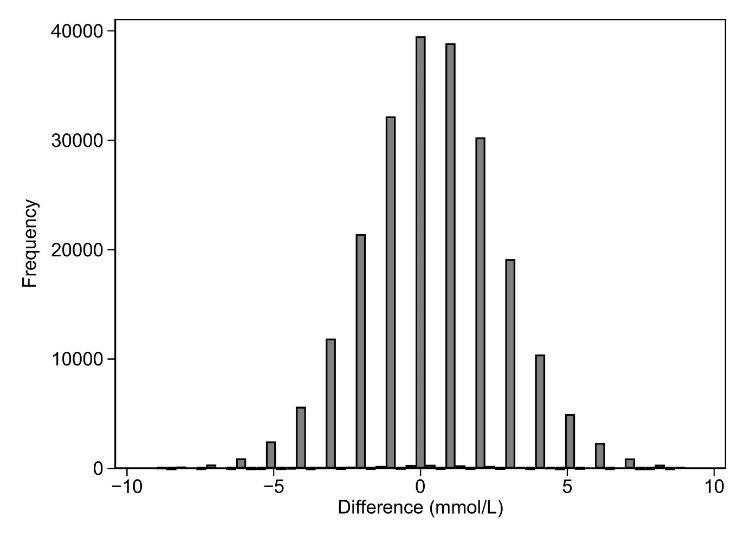


*Figure 1. Frequency histogram of the numerical value of calculated difference between laboratory sodium and POC sodium. A difference of 0mmol/L was the most common value.*

Table 1. Sample predictions

| Sodium range | ABG Na | Lab Na (Deming) | Lab Na (OLS) |
| --- | --- | --- | --- |
| All | 140.0 | 140.4 | 139.3 |
| Hyponatraemia | 125.0 | 122.5 | 127.6 |
| Eunatraemia | 140.0 | 140.2 | 138.9 |
| Hypernatraemia | 155.0 | 154.0 | 151.7 |

Table 2. Intraclass correlation coefficients (absolute agreement) for all data and hyponatraemia, eunatraemia, and hypernatraemia subgroups, with comparison of values in individual samples, and the average values.

| Sodium range | ICC (Individual) | ICC (Average) | p-value |
| --- | --- | --- | --- |
| All data | 0.834 (0.819, 0.847) | 0.910 (0.901,0.917) | < 0.001 |
| Hyponatraemia | 0.688 (0.362, 0.824) | 0.815 (0.531, 0.904) | < 0.001 |
| Eunatraemia | 0.647 (0.641, 0.652) | 0.786 (0.781, 0.790) | < 0.001 |
| Hypernatraemia | 0.523 (0.313, 0.659) | 0.687 (0.477, 0.794) | < 0.001 |

Table 3. Mean difference in each subgroup

| Sodium range | Description | N | Mean difference (95% LoA^a^) |
| --- | --- | --- | --- |
| Na < 120 | Severe hyponatraemia | 535 | +2.01 (8.08) |
| 120 ≤ Na < 130 | Moderate hyponatraemia | 7978 | +1.99 (4.51) |
| 130 ≤ Na < 135 | Mild hyponatraemia | 48418 | +1.45 (4.08) |
| 135 ≤ Na ≤ 145 | Eunatraemia | 149469 | +0.15 (4.37) |
| 145 < Na ≤ 150 | Mild hypernatraemia | 11897 | -0.77 (4.88) |
| 150 < Na ≤ 160 | Moderate hypernatraemia | 5928 | -1.42 (5.57) |
| Na > 160 | Severe hypernatraemia | 158 | -4.70 (15.46) |
| All data | Total | 224383 | +0.72 (4.35) |

Table 4. Orthogonal least squares modelling for comparison to Deming regression.

| Sodium range | Slope (b) | p-value | Intercept (a) |
| --- | --- | --- | --- |
| All | 0.78 (0.77, 0.79) | < 0.001 | +30.14 |
| Hyponatraemia | 0.91 (0.90, 0.92) | < 0.001 | +13.82 |
| Eunatraemia | 0.74 (0.73, 0.75) | < 0.001 | +35.31 |
| Hypernatraemia | 0.69 (0.63, 0.74) | < 0.001 | +44.76 |

Table 5. NaL was tested against NaP. χ2 values are given for each group with degrees of freedom in brackets. Symmetry was assessed with McNemar-Bowker Test and homogeneity was tested using the Stuart-Maxwell test.

| Sodium range | Asymptotic symmetry | Marginal homogeneity | p-value |
| --- | --- | --- | --- |
| All data | 4667 (595) | 4204 (124) | < 0.001 |
| Hyponatraemia | 9569 (276) | 9126 (68) | < 0.001 |
| Eunatraemia | 5151 (257) | 5015 (50) | < 0.001 |
| Hypernatraemia | 569 (134) | 519 (46) | < 0.001 |

*Table 6. Multivariable regression of common biochemical markers.*

| Predictor | β (95% CI) | p-value |
| --- | --- | --- |
| pH^1^ | +0.133 (+0.120, +0.145) | < 0.001 |
| Chloride | -0.027 (-0.028, -0.025) | < 0.001 |
| Lactate | -0.009 (-0.016, -0.002) | 0.011 |
| Urea | -0.0002 (-0.0016, +0.0013) | 0.828 |
| Albumin | -0.048 (-0.049, -0.046) | < 0.001 |

*^1^ For pH, the regression coefficients were calculated for increments of 0.1 pH units.*

*Table 7. Sensitivity analysis with comparison of mean difference across all data, compared to mean difference in one random paired sample per patient.*

| Sample | Mean (SD) | p-value |
| --- | --- | --- |
| All data | +0.72 (2.22) |  |
| Random sample per patient | +0.17 (2.40) | P<0.001 |
